# Design and Performance Study for Electrothermally Deep-Sea Drive Microunits Using a Paraffin Phase Change Material

**DOI:** 10.3390/mi12040415

**Published:** 2021-04-09

**Authors:** Dayong Ning, Zihao Li, Gangda Liang, Qibo Wang, Weifeng Zou, Yongjun Gong, Jiaoyi Hou

**Affiliations:** 1 National Center for International Research of Subsea Engineering Technology and Equipment, Dalian Maritime University, Dalian 116026, China; ningdayong@dlmu.edu.cn (D.N.); lzhmvp@foxmail.com (Z.L.); lianggangda@dlmu.edu.cn (G.L.); wqb201908@dlmu.edu.cn (Q.W.); zwf.maple@foxmail.com (W.Z.); gyj@dlmu.edu.cn (Y.G.); 2 State Key Laboratory of Fluid Power and Mechatronic Systems, Zhejiang University, Hangzhou 310027, China

**Keywords:** paraffin, phase change material, deep-sea, microunit

## Abstract

Considering the further exploration of the ocean, the requirements for deep-sea operation equipment have increased. Many problems existing in the widely used deep-sea hydraulic system have become increasingly prominent. Compared with the traditional deep-sea hydraulic system, actuators using a paraffin phase change material (PCM) have incomparable advantages, including lightweight structure, low energy consumption, high adaptability to the deep sea, and good biocompatibility. Thus, a deep-sea drive microunit (DDM) based on paraffin PCM is proposed in this paper. The device adopts a flexible shell, adapting to the high-pressure environment of the deep-sea based on the principle of pressure compensation. The device realizes the output of displacement and force through the electrothermal drive, which can be used as actuator or power source of other underwater operation equipment. The microunit successfully completes the functional verification experiments in air, shallow water, and hydrostatic pressure of 110 MPa. In accordance with experimental results, a reasonable control curve is fitted, highlighting its potential application in deep-sea micro electro mechanical systems, especially in underwater soft robot.

## 1. Introduction

The ocean is rich in resources, such as minerals, biology, and seawater. These resources promote the development of society and are a new space for human survival and development. The importance of the ocean is self-evident [1,2,3,4]. With the increasing degree of exploration and exploitation of marine resources, marine exploration is gradually moving forward from the shallow layer to the deep layer, and the depth and breadth of exploration are increasing. The advanced underwater operation equipment is a powerful tool for the development of marine resources. However, different from shallow sea development, extreme environments, such as deep-sea high pressure and low temperature, put forward high requirements for the performance of underwater operating equipment [5].

At present, because of its mature technology, design, and manufacturing experience, the hydraulic system has become the preferred drive and control mode for generations of underwater equipment designers. In the deep-sea complex marine environment, the deep-sea hydraulic system uses mineral oil as a working medium. Moreover, some problems, such as environmental pollution (the working medium is not compatible with the marine environment), complex structure (needs pressure compensation, fuel tank, return pipe), and poor working reliability (strict sealing requirements; seawater intrusion into the system causes oil deterioration, aggravation of corrosion, and wear of components), are observed [6]. Research on seawater hydraulic technology has been carried out successively to adapt to the deep-sea environment and meet the needs of large-depth and large-scale underwater operations. Although the seawater hydraulic system has incomparable advantages over the oil hydraulic system, practice has proven that the complete use of existing methods cannot fully adapt to the special working conditions of the deep sea. The deep-sea environment is extremely changeable, and the maximum environmental pressure is 110 MPa. The physical and chemical properties of the working medium of the hydraulic system, such as density, viscosity, and bulk elastic modulus, change with the environment, which has a considerable impact on the dynamic and static performances of the components and the system [7]. Many friction pairs of system components and the scale, direction, and consistency of their deformation vary remarkably under the combined action of deep-sea temperature and pressure. Seawater is highly corrosive, which brings evident corrosion and wear problems [8]. Moreover, microbial corrosion in seawater hydraulic components occurs because of the large amount of suspended sediment in the natural seawater medium, thereby putting forward new requirements for the design of a seawater hydraulic system [9,10,11,12].

In recent years, scientists have explored some novel driving methods, such as phase change driving. The change in material volume or shape caused by phase transformation is one of the most important driving forces behind the driver. For example, Gill et al. used microgrippers made of shape memory alloy, which are triggered electrically and can be used for grabbing microobjects in a living organism [13]. Ikeda et al. used crosslinked liquid crystal elastomer (CLCP) films as light-driven actuators to control the bending direction of CLCP films by changing the polarization direction of incident light; this mode of deformation is very advantageous for an artificial “hand” [14]. Ma et al. developed a multifunctional hydrogel microactuator by combining a thermally responsive graphene oxide GO-PNIPAM sheet and a pH-responsive fluorescent hydrogel sheet. The hydrogel microactuator changes its shape with temperature and its color with pH [15]. The above devices have achieved lightweight design but are evidently not suitable for underwater or deep-sea working conditions. Moreover, these devices cannot produce enough force to meet the requirements of the task. We expect to produce force in the range of approximately 1 to 3 kgf.

Ogden et al. compared the energy densities of different actuator materials and found that phase change materials (PCMs) have the highest energy density compared with shape memory alloys and electromagnetic, thermopneumatic, bimetallic, electrostatic, and piezoelectric actuator materials [16]. The high energy density is due to the high thermal expansion coefficient and low compressibility [17]. Paraffin is activated by applying heat. The activation time depends on the time required for heat supply to and distribution within the paraffin [18,19,20]. Paraffin has a significant volume increase when transformed from the solid state to the liquid state. According to Zoller and Srivastava, the volume of paraffin can vary as much as 43.5% when the temperature goes from 30 °C to 250 °C at a pressure of 0 MPa. Its small compressibility shows its potential as a suitable PCM, and the expansion is retained to a large extent, even at pressures up to 200 MPa [17,21]. This feature is also the prerequisite to realize the transmission of large torque in a small volume [22,23]. The high thermal expansion coefficient and low compressibility of paraffin provide a guarantee for the performance of the microunit designed and manufactured in this paper.

At present, micro phase change actuators made of paraffin are applied in many fields. The stainless-steel high-pressure membrane microvalve made by Sharma et al. can withstand back-pressures of 200 bar with response time of less than 0.6 s [24]. Moreover, this microvalve structure is simple, reducing the drawbacks of traditional valves at run time [25,26]. A Micro Electro-Mechanical Systems louver was used to achieve a thermal control function for spacecraft and instruments. In the description of the device, PCMs, such as paraffin, were used to open and close mechanical vanes or windows to allow an alterable radiative view to space [27]. Lee et al. produced electrothermal hydraulic microactuators by using paraffin. These microactuators were integrated into microfluidic valves, microgrippers, and micropipettes, and their performances are demonstrated [28]. Ayers et al. designed a paraffin-actuated micromirror and realized the movement of the mirror in a spiral-like fashion, which is successfully applied in gastrointestinal imaging [29]. Hou et al. developed an electrothermally driven deep-sea buoyancy control module, which has been successfully verified at 3223 m to the seabed of the South China Sea. This device also uses the volume change generated by paraffin in the process of phase transformation [30].

Inspired by this, a phase change-driven microunit with flexible material as shell is designed in this paper. The microunit uses an electrothermal driver to control heating and cooling of the paraffin. This microunit can serve as an actuator or power source for various underwater equipment, which can replace the traditional deep-sea hydraulic system. Based on the principle of pressure compensation, the seawater pressure is sensed using an elastic shell and transferred to the interior of the microunit to avoid the influence of sea water pressure [1,31]. Compared with the existing deep-sea hydraulic system, this microunit remarkably reduces the volume and weight, simplifies the difficulty of manufacturing, eliminates the influence of friction and corrosion, and reduces the consumption of energy. These features profoundly reflect the microunit’s potential application in the deep-sea micromechanical system.

As the basis of this study, the displacement characteristics of the microunit driven by the electric heating wire are observed using a micro-laser displacement sensor. Immersed in cold water, the effect of ambient temperature on its displacement is observed. Part of the driving force is measured by the force sensor and is placed in a special pressure chamber to verify the working ability of the microunit designed in this paper under high hydrostatic pressure. Theoretical analysis and experiments show a good functional relationship between the displacement change of the microunit and the heating time, indicating that the device can accurately control displacement and achieve the predetermined function in a high-pressure environment.

## 2. Design and Fabrication

### 2.1. Design

**Figure 1:**
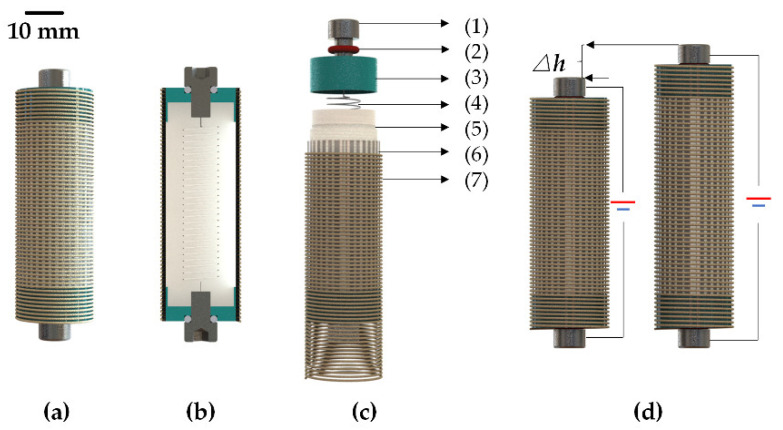
Electrothermal deep-sea drive microunit: (**a**) Overall schematic, where *h* is the height of prototype shown in Table 1, (**b**) schematic cross-section showing the main internal structure, where *r* is the radius of prototype and *b* is the thickness of SR shell shown in Table 1, (**c**) exploded view showing the various components and combinations of the microunit, i.e., (1) electrode, (2) high temperature-resistant O-ring, (3) end cover, (4) heating wire, (5) paraffin PCM, (6) SR shell, (7) restricted layer. (**d**) Working principle diagram of the microunit, where Δ*h* is the displacement shown in Table 1.

The design goals are to build a system that is lightweight, miniature, and suitable for a deep-sea environment. The design scheme used silicone rubber (SR) as matrix to prepare shell to wrap the paraffin and internal components. This structure can ensure the high-pressure resistance of the deep-sea drive microunit and realize its working ability in the deep-sea environment. In addition, the shell, paraffin, and internal components were ensured to not have air gaps, and degassing and continuous refilling were chosen in accordance with this. The generation of displacement and output force in the deep-sea drive microunit comes from the large and rather incompressible volume expansion associated with the solid-to-liquid phase transition of phase change materials. According to previous studies by scholars at home and abroad, the volume change near the melting point of paraffin phase change is about 17%, and the volume expansion can be retained to a large extent when the pressure is as high as 200 MPa [17,21]. Therefore, the selection of paraffin as phase change material can ensure that the microunit can still deliver large strokes and high actuation forces at small scales. Figure 1 shows the structure, components, and working principle of the deep-sea drive microunit to describe the design scheme and working principle of the proposed device.

Considering the laboratory manufacturing level and its own experience, the design parameters of the deep-sea drive microunit are shown in Table 1.

The working principle of the microunit is when the paraffin in the microunit is heated by the heater, increased temperature and phase change are observed, bringing nearly incompressible free volume expansion. Under the action of the external limiting layer, the volume expansion is limited in the axial direction for the microunit to produce axial elongations and actuation forces. Therefore, the relationship of axial displacements can be obtained as follows:

(1) $$\Delta h=\frac{\alpha \cdot V_{PCM}}{\pi \cdot {\left(r-b\right)}^{2}}$$

The parameters in the formula are consistent with those in Table 1.

### 2.2. Fabrication

The deep-sea drive microunit was fabricated using the modular integrated process, which was beneficial to the batch production of the unit. The following described the components of each part one by one according to the sequence of the preparation process.

The elastic shell was formed by casting in a mold (Figure S1). After designing with Solidworks, the mold was printed using the 3D printer (UnionTech Lite 300, Shanghai, China) by StereoLithography Apparatus. The shell was made of AB two-component liquid silicone rubber of Shore hardness A15 mixed at a 1:1 weight ratio. The mixture was placed into a vacuum chamber for degassing for 15 min to remove bubbles, then poured into the mold, and placed in a drying oven for high-temperature curing for 8 h. The silicone rubber had good heat resistance, cold resistance, electrical insulation, flexibility, ductility, and biocompatibility. Its work temperature ranged from −30 °C to 200 °C. These characteristics were conducive to its work in deep sea with high corrosion, high electrical conductivity, and low temperature, and ensure the sealing of paraffin. At the same time, it had a long curing time at room temperature, which provided sufficient time to remove the air in silica gel during production.

The upper and lower end covers were made of nylon material printed using a 3D printer (HP Jet Fusion 4200, Palo Alto, CA, USA) by MultiJet Fusion. The material can maintain the stability of its original properties at 175 °C temperature. The screw hole ensured the fixation of the bolt, and the O-ring placed under the countersunk head of the bolt ensured the good sealing of the microunit body. These two bolts acted as a fixing frame of the electric heating wire and as positive and negative electrodes of the microunit.

The material of the electric heating wire inside the microunit was Cr20Ni80 alloy. A winding machine was used to make the nichrome wire into a regular spiral structure and distributed symmetrically in paraffin to improve the heat conduction efficiency in paraffin. The use of a winding mechanism as electric heating wire can ensure the consistency of the resistance, which was beneficial to the simultaneous control of multiple microunits in the future. The end cover, bolts, and electric heating wire were assembled and bonded to the elastic shell with a 704-silicone rubber.

The paraffin inside the microunit was made by casting. The 58# fully refined paraffin was selected here. This type of paraffin was a mixture of alkanes and was an inexpensive, nontoxic, and harmless material. Paraffin was melted by heating before casting and placed into a vacuum chamber for degassing for 15 min. In the process of degassing, if the paraffin changed into a molten state due to decreased temperature, it should be taken out, melted by heating, and placed into a vacuum chamber for degassing. After the degassing process was completed, the paraffin was slowly injected into the elastic shell bonded in the previous step. During cooling, additional paraffin was poured into the shell to compensate for the volume lacking in the shell due to shrinkage. Filling the shell in its entirety was achieved by continuous refilling up to a surplus.

The outer limiting layer was surrounded by the fine winding of aramid fibers. The aramid fiber is a new type of high-tech synthetic fiber; has excellent properties, such as ultrahigh strength, high modulus, acid and alkali resistance, lightweight, and wide temperature; and is suitable to work in deep sea [32]. Such winding squeezed out the air that remained between the inner wall of the microunit and the outer wall of the paraffin. The radial displacement of the microunit can be limited. Thus, the displacement output only exists in the axial direction.

**Figure 2:**
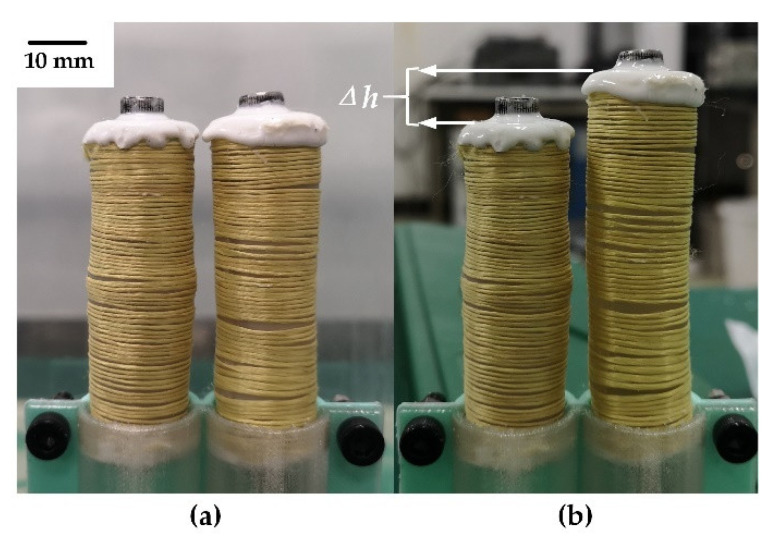
Theoretical and functional verification experiments: (**a**) original state and (**b**) state after displacement of Δ*h*.

After a sample was prepared, theoretical and functional verification experiments were carried out first. As shown in Figure 2, the experimental results showed that the microunit can complete the desired stroke and reach displacement of 8.62 mm. Moreover, no leakage of paraffin or any other problem was observed during the experiments.

## 3. Experimental Procedures

As an actuator, reliable and controllable displacement characteristics are key to measuring performance and working capacity. Therefore, experiments on the displacement characteristics of a deep-sea drive microunit are designed to be carried out in two working environments, i.e., air at 18 °C and shallow water at 6 °C, using different power values. The mechanical properties of the actuator and the magnitude of the force produced directly determine its working range. Based on previous design requirements and the special working environment of the deep sea, a high-pressure resistance experiment is designed to verify the functional verification of the microunit under a high-pressure working environment. All the above experiments were repeated five times.

### 3.1. Displacement Characteristics Test

#### 3.1.1. Air Displacement Characteristics Test

**Figure 3:**
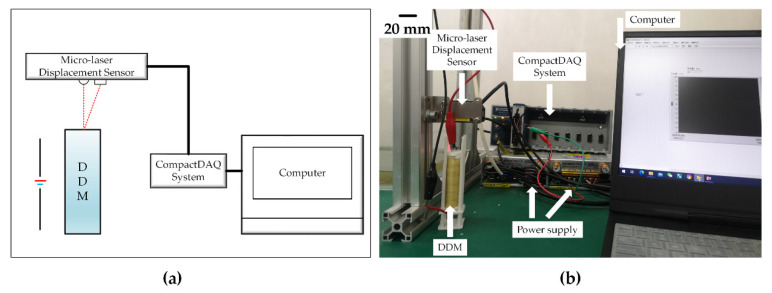
Air displacement characteristics test bench: (**a**) Schematic of air displacement characteristics testing system, and (**b**) air displacement characteristics test site.

The experimental system for testing the displacement characteristics of the deep-sea drive microunit in air at 18 °C is shown in Figure 3. The experimental equipment included a micro-laser displacement sensor (Panasonic HG-C1030, Suzhou, Jiangsu, China; measuring range = 10 mm, repetition accuracy = 10 μm), a prepared deep-sea drive microunit, a holder, a 0–48 V adjustable DC power supply, the NI CompactDAQ, and a computer. The PC program was written by LabVIEW to monitor and record the displacement data of micro-laser sensor collected by the NI CompactDAQ in real time. The deep-sea drive microunit was placed in the holder to prevent the microunit from tilting during deformation. The micro-laser displacement sensor was arranged at 25 mm from the upper part of the microunit and fixed on an aluminum profile with a holder and bolt to maintain its position. The power of the heating wire was controlled using an adjustable power supply. Next, the data collected by the micro-laser displacement sensor were transmitted to the NI Compact-DAQ and transferred to a computer for recording, classification, and analysis. Table 2, Test No. 1 shows several important parameters of the experimental conditions.

#### 3.1.2. Underwater Displacement Characteristics Test

**Figure 4:**
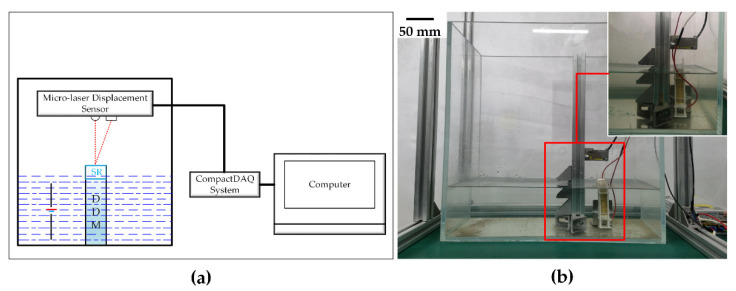
Underwater displacement characteristics test bench: (**a**) Schematic of underwater displacement characteristics testing system, and (**b**) underwater displacement characteristics test site.

Experiments on displacement characteristics were designed in low temperature and shallow water. The experimental system to test the displacement characteristics of the deep-sea drive microunit in shallow water at 6 °C is shown in Figure 4. Considering the special characteristics of the underwater and the deep-sea environment, the positive and negative poles of the deep-sea drive microunit were waterproof and encapsulated with 704-silicone rubber before the underwater experiment. The deep-sea environment has high corrosion and high electrical conductivity. The experiments in South China Sea show that the bonding of 704-silicone rubber can be applied to the deep-sea environment, which can ensure that the wrapped metal electrode is not negatively affected by seawater [30]. Given the refraction of laser in water, the micro-laser displacement sensor cannot effectively and accurately measure the displacement characteristics of the deep-sea drive microunit in water. Therefore, a cylinder was poured using 704-silicone rubber at the top of the deep-sea drive microunit. In the experimental process, immersion of the entire deep-sea drive microunit in water was ensured, and the secondary pouring section above water as a platform was maintained for focus projection. The other conditions were consistent with the experiment in the air environment. Table 2, Test No. 2 shows several important parameters of the experimental conditions.

### 3.2. Force Characteristics Test

**Figure 5:**
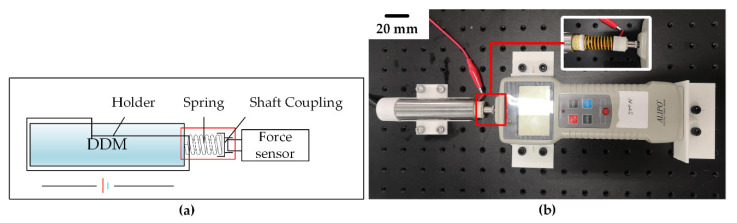
Force characteristics test bench: (**a**) Schematic of the force characteristics testing system, and (**b**) force characteristics test site.

The deep-sea drive microunit produces thrust while elongating. According to this feature, an experimental system was designed to test its mechanical properties. The principle and experimental equipment of the thrust test system are shown in Figure 5. Through this system, the force which can be output by the deep-sea drive microunit in the stroke direction can be measured. Given that the shell of the microunit was flexible silicone rubber material, paraffin produced free volume expansion. The limiting layer only limited its radial expansion and allowed it to produce axial elongation. If the microunit is directly used to push the external object during elongation, the microunit will produce a certain bending deformation. The axial force, which can be output, cannot be obtained accurately. Therefore, the microunit was placed in a steel pipe whose inner diameter was almost the same as the microunit’s outer diameter. The inner wall is smooth to ensure that the direction of elongation after heating is kept in the axial direction and will not be changed by external forces. Then, the steel pipe was fixed on a custom holder. On the right, a spring with 12.75 N/mm rigidity was installed between the deep-sea drive microunit and the force sensor through a connecting device. The spring can buffer the displacement of the microunit, protect the experimental device, and ensure the normal operation of the experiment. The force sensor measured the axial force and friction force output by the microunit. Given that the steel pipe inner wall is relatively smooth, the indicator of the dynamometer can be approximately equal to the axial force output by the microunit. Table 2, Test No. 3 shows several important parameters of the experimental conditions.

### 3.3. Anti-Pressure Characteristics Test

**Figure 6:**
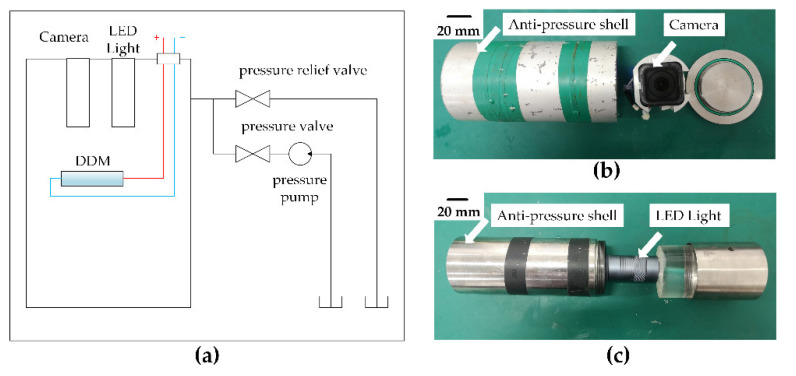
Anti-pressure characteristics test bench: (**a**) schematic of the anti-pressure characteristics testing system, (**b**,**c**) experimental processes recorded using a video camera (**b**), and LED light (**c**) protected by anti-pressure shells.

The requirement for a lightweight and miniature deep-sea drive microunit is that it is free of any bulky or heavy anti-pressure system and ensures the flexibility of the microunit in use. Some pieces of equipment used in the experiment are shown in Figure 6. The whole experiment was carried out in a special pressure chamber. The high-pressure hydraulic pump pumped water into a pressure chamber with an inner diameter of 80 cm and height of 2 m to simulate the 110 MPa condition in deep sea. The power supply was supplied to the deep-sea drive microunit placed in the pressure chamber through the deep-sea cable watertight joint. Its displacement characteristics are described by the steel ruler placed below. Experimental processes were recorded using a video camera and LED light protected by anti-pressure shells. Table 2, Test No. 4 shows several important parameters of the experimental conditions.

## 4. Results

### 4.1. Displacement Characteristics

#### 4.1.1. Air Displacement Characteristics

**Figure 7:**
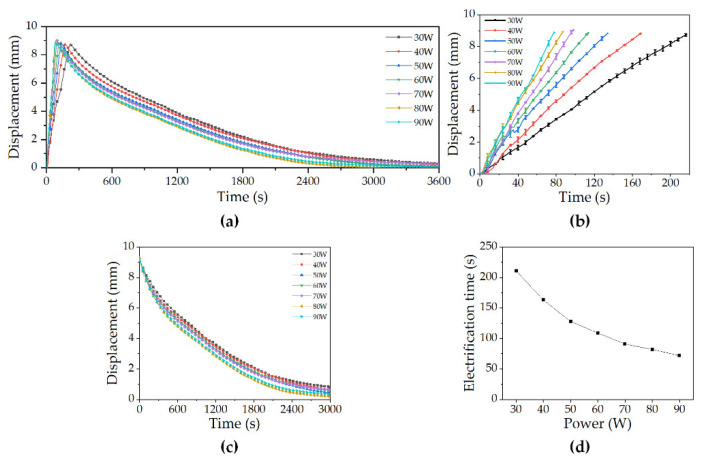
Experimental results of DDM in air: (**a**) Displacement change monitoring of the DDM under different power values, (**b**) displacement change monitoring of DDM running under different power values, (**c**) displacement change data of the microunit during working withdrawal within 3000 s after stopping heating, and (**d**) response time of DDM in air.

The displacement characteristics experiment in air verified that the designed and manufactured deep-sea drive microunit had controllable and reliable displacement characteristics. Figure 7a shows the monitoring data of the complete process of the seven groups of experiments under different power values. Figure 7b shows that the microunit can reach and exceed the predetermined displacement of 17%, i.e., 8.62 mm. Moreover, the recorded maximum value was 9.03 mm. Upon reaching the recorded maximum value, the trend was still consistent with the previous trend. Moreover, no growth attenuation phenomenon was observed. This result showed that the recorded maximum value was far from reaching the displacement limit of the microunit. The test was carried out within the range of the predetermined design index to ensure safety and avoid the thermal fatigue of the end cover caused by overheating of the paraffin. As shown in Figure 7b, the displacement change data of the microunit during elongation are extracted. A strong linear correlation between the electrification time and the displacement of the microunit under different power values was observed. This result indicated that the microunit was expected to have the same controllability as the traditional actuator. As shown in Figure 7c, the displacement change data of the microunit during the retraction process within 3000 s after heating is stopped are extracted. In this experiment, the retraction of microunit depended on the natural convective heat transfer cooling in air. The active heat dissipation structure was not added to simplify the structure and volume of the microunit as much as possible and adapted to the condition of deep-sea high-pressure.

Figure 7d shows the response time of the microunit (Table S1). The electrification time was the time when the displacement reached 8.62 mm. When the power reached 90 W, the predetermined displacement of 17% was reached after 72 s. The average retraction time was the time it took to recover almost its original length. In this group of experiments, the average retraction time was 3000 s.

#### 4.1.2. Underwater Displacement Characteristics

**Figure 8:**
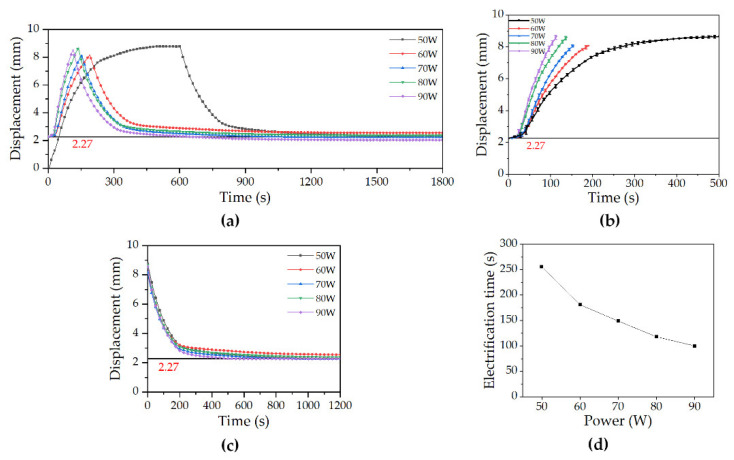
Experimental results of DDM underwater: (**a**) Displacement change monitoring of the DDM under different power, (**b**) displacement change monitoring of DDM running under different power, (**c**) displacement change data of the microunit during working withdrawal within 1200 s after stopping heating, and (**d**) response time of DDM underwater.

In the experiment of displacement characteristics in air, the displacement characteristics of microunit were reliable and controllable. This shows that the microunit can work effectively in air and complete specific tasks. Next, the displacement characteristics were tested in low temperature and shallow water. In the water tank, the microunit still worked under different power values. Figure 8a shows the monitoring data of the complete process of five groups of experiments under different power values. As shown in Figure 8b, the displacement change data of the microunit during elongation are extracted. Unlike in air, the displacement change rate decreased with increasing displacement, thereby proving that the heat exchange in water was the main factor affecting its performance. As shown in Figure 8c, the displacement change data of the microunit during the retraction process within 1200 s after the heating is stopped are extracted. The water temperature environment at 6 °C remarkably accelerated the cooling and retraction of the microunit. However, this environment also led to the residue of 2.27 mm displacement.

Figure 8d shows the response time of the microunit (Table S2). The electrification time referred to the response time of the specific displacement interval of 2.27–8.62 mm. The average retraction time was the response time when the maximum value returned to 2.27 mm in each group of experiments. In this group of experiments, the average retraction time was 190 s.

### 4.2. Force Characteristics

The deep-sea drive microunit produces thrust while elongating. The output force is extremely large due to the nearly incompressible volume expansion caused by the phase change [17]. After the experiments, the microunit’s working ability was 3.53–3.82 kgf. The average value of multiple experiments was 3.62 kgf, which met the expected design index. Therefore, we did not continue to experiment to explore its greater working ability. However, when the data were obtained, the silicone rubber shell and aramid fiber of DDM were undamaged. Then, the sample was tested for its air displacement characteristics at 90 W power, and it was found that its displacement characteristic curve was not affected. This showed that the sample can continue to work normally.

### 4.3. Anti-Pressure Characteristics

**Figure 9:**
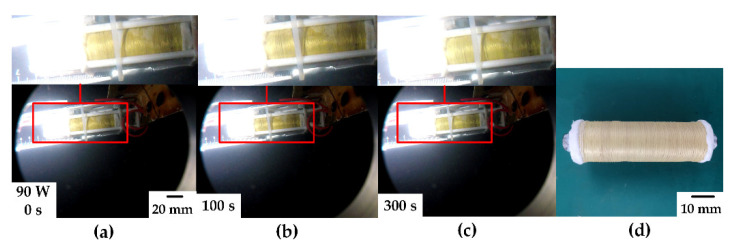
DDM test at 110 MPa hydrostatic pressure: (**a**–**c**) experimental process at 90 W and (**d**) prototype at 110 MPa hydrostatic pressure.

The experimental results of DDM underwater verified that the DDM prototype produced by us had controllable displacement characteristics. Next, the anti-pressure test was carried out. First, in the pressure chamber, a set of preparatory experiments was carried out at 17.8 °C and 10 MPa hydrostatic pressure (Figure S2). The microunit took 240 s to complete the displacement of 6 mm successfully at 50 W power and returned to a state close to its original length after about 260 s. After the abovementioned experiments, the samples were collected and checked. The ways of checking included observation and reheating test. After checking, the shape of microunits was able to retain its original state, with no damage and leakage of paraffin. This showed that the microunit can work normally at 10 MPa hydrostatic pressure. Then, the pressure chamber was placed again. Under hydrostatic pressure of 110 MPa, several groups of experiments were carried out with different power values. As shown in Figure 9a–c, an experimental picture with a power of 90 W was selected. It can be seen from the comparison in the figure that the microunit can still show evident displacement and successfully complete the expected action under hydrostatic pressure and temperature of 110 MPa and 17.8 °C, respectively. Figure 9d shows one of the prototypes under high pressure of 110 MPa. Combined with the results in Figure 9d and the performance in 110 MPa experiments, this design can fully withstand the hydrostatic pressure of 110 MPa. Under such conditions, the microunit can also complete the expected function. This further validated the pressure-resistant design strategy in this paper and achieved a lightweight design.

### 4.4. Displacement Characteristics Curve Fitting Analysis

From the above experimental results, the microunit was found to be able to achieve the expected volume expansion under high pressure of 110 MPa, which can be used as an actuator or power source of other underwater equipment. Moreover, the realization of this process only needed to provide enough heat to the microunit. The heat can be provided by a portable battery without other mechanical structures. This gave the whole system the advantages of simple form, small size, and light weight. Experimental results show that the microunit had different displacement characteristics in air and cold water but a relatively clear functional relationship, which makes it possible that the microunit is close to or even has the same controllability as the traditional drive system.

**Figure 10:**
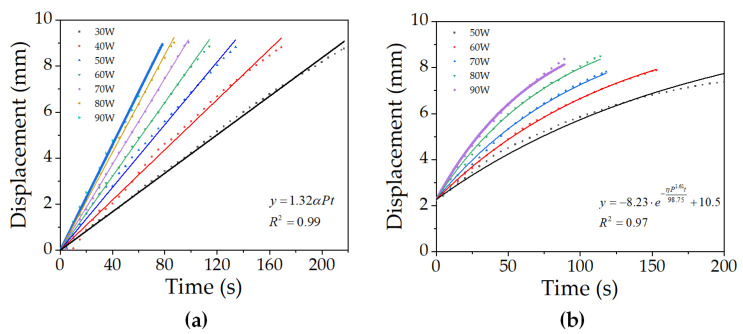
Data postprocessing: curve fitting in the ascending phase (**a**) in air and (**b**) underwater.

Then, the experimental data of input power less than 90 W in air and cold water are processed, as shown in Figure 10.

On the basis of the numerical analysis of the experimental results in air, the fitting formula of the effect of input power and electrification time on the displacement of the microunit was obtained as follows:

(2) y=1.32λPt

The *R*-squared of this fitting model is 0.99. *y* is the displacement (m), *λ* is the coefficient (1 × 10^−6^ $\frac{s^{2}}{\mathrm{kg}\cdot m}$), *P* is the input power (W), and *t* is the electrification time (s).

Based on the numerical analysis of the experimental results in cold water, the fitting formula of the effect of input power and electrification time on the displacement of the microunit was obtained as follows:

(3) $$y=-8.23\cdot e^{-\frac{\eta P^{1.61}t}{98.75}}+10.5$$

The *R*-squared of this fitting model is 0.97. *η* is the coefficient, (1 × 10^3^ $\frac{s^{2}}{\mathrm{kg}\cdot m}$).

Finally, three groups of data with input power of 90 W were randomly selected to verify the fitting formula. The percentage error is shown in Table 3. The results show that the error is about 6%. The calculation results of the fitting equation can well fit the experimental data.

## 5. Discussion

### 5.1. Air Displacement Characterization

As shown in Figure 7, the front end of the 50 W power curve was not linear. This was because the microunit tilted during deformation. If the microunit tilts during deformation, the measurement focus of the micro-laser sensor cannot be projected to the correct position. Thus, the micro-laser sensor cannot really reflect the displacement characteristics of the microunit, thereby resulting in measurement errors. The existence of measurement errors also led to the decrease of goodness of fit, which was reflected in the decrease of *R^2^* in Figure 10. The tilt of the deep-sea drive microunit during deformation was due to two reasons. First, the electric heating wire arranged inside the unit body may have been slightly skewed during the fabrication process. Second, in the test process, the deep-sea drive microunit was subjected to the external force brought about by the crocodile clamp at the top. Among the two reasons, the second was the main factor. After testing multiple microunits made in different batches, the effect of the first reason was not remarkable. Therefore, the requirement of neutrality in the arrangement of heating wire was not extremely high in the manufacture of this microunit. This phenomenon also reduced the difficulty of production and is conducive to the realization of mass production.

### 5.2. Underwater Displacement Characterization

Unlike the data presented in Figure 7d, the microunit’s electrification time was extended due to the external low temperature water environment. However, low temperature remarkably reduced its average retraction time, further verifying the adaptability of microunit in deep sea. On the other hand, the low-temperature environment also led to the generation of residual displacement. After analysis, the generation of this residual displacement was related to the elastic modulus of the silicone rubber shell and the curing rate of the paraffin outer wall. When the elastic modulus of the silicone rubber shell increased or the curing rate of the paraffin outer wall decreased, the length of this residual displacement was reduced.

**Figure 11:**
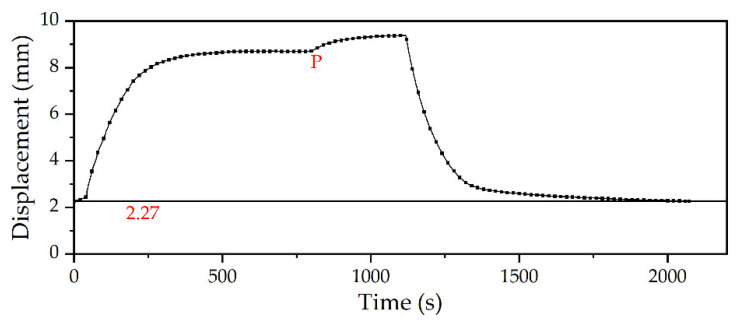
Supplementary experiment of residual displacement.

From the results of the underwater displacement characteristics test, the displacement characteristic curve with power of 50 W was significantly different from that of the other groups. Thus, the experiment was carried out to determine the reason (Figure 11). At the beginning, 50 W power was used to heat for 800 s to reach point P, and the power was increased to 60 W. After evident displacement changed, the power supply was turned off. It can be found from the figure that when the microunit elongated to 8.7 mm, that was, when the microunit was 78.7 mm as a whole, it was no longer elongated under the power value and ambient water temperature of 50 W and 6 °C, respectively. However, when the power increased to 60 W, the microunit continued to elongate the displacement. This result showed that, in this case, the microunit reached the thermal equilibrium state with the external environment but did not reach the limit of its displacement. The emergence of this phenomenon enhanced the controllability of the microunit, making it possible for the microunit to achieve quantitative elongation and maintain a stable state to complete tasks.

### 5.3. Force Characterization

Although the phase change can produce incompressible volume expansion, this can output extremely large force. For this microunit, the output force was limited by the strength of the silicone rubber shell and the aramid fiber. In the experiment, the magnitude of the DDM working ability was also affected by the stiffness of the adopted spring and the non-axial deformation of the microunit. According to the research of Mann et al., the substantial potential space for the microunit needs to be developed and can be further explored and improved upon [23].

## 6. Conclusions

An electrothermally flexible deep-sea drive microunit was designed, manufactured, and evaluated. Experimental studies, functional test, and performance test of the system reveal that the designed and manufactured deep-sea drive microunit has good functional characteristics. The tests in this paper show a good functional relationship between the change in displacement, input power, and electrification time. Fitting formulas are then obtained. This shows that it can approach or even achieve the same controllability as the traditional deep-sea drive system. Compared with the traditional deep-sea drive system, the microunit has excellent lightweight performance, high pressure resistance, and energy-saving feature. This means that an autonomous or semiautonomous micro deep-sea soft robot can be constructed. Further research is needed on the residual displacement, maximum output force, and control methods of microunits in deep sea high-pressure environments. Thus, the robot of microunits modular combination can perform diverse tasks by precisely adjusting the displacement in the desired direction.

## Figures and Tables

**Table 1:** Parameters of the electrothermal deep-sea drive microunit.

Parameter	Symbol	Values
Mass of prototype	*M_DDM_*	12,908.49 mm^3^
Mass of filled paraffin PCM	*M_PCM_*	11.62 g
Volume of paraffin PCM	*V_PCM_*	30 g
Height of prototype	*h*	70 mm
Radius of prototype	*r*	10 mm
Thickness of SR shell	*b*	1 mm
Displacement	Δ*h*	8.62 mm
Resistance of heating wire	*R*	9 Ω
Volume expansion rate of PCM	*α*	17%

**Table 2:** Experimental conditions.

Test No.	Test Environment	Environment Temperature (°C)	Environment Pressure (MPa)	Power (W)
1	Air	18	0.1	30, 40, 50, 60, 70, 80, 90
2	Water	6	0.1	50, 60, 70, 80, 90
3	Air	18	0.1	/
4	Water	17.8	110	50, 60, 70, 80, 90

**Table 3:** Percentage error of displacement.

Test Environment	Power (W)	Electrification Time (s)	Percentage Error (%)
Air	90	30 50 70	−0.40% 5.51% 2.64%
Underwater	90	35 55 65	1.25% 0.86% −0.15%

## Supplementary Materials

The following are available online at https://www.mdpi.com/article/10.3390/mi12040415/s1, Figure S1: Fabrication process of DDM, Figure S2: DDM test at 10 MPa hydrostatic pressure, Table S1: Response time of DDM in the air, Table S2: Response time of DDM underwater.
