# Supplementary material for: Design and Performance Study for Electrothermally Deep-Sea Drive Microunits Using a Paraffin Phase Change Material
Source: Micromachines (Basel). 2021 Apr 9;12(4):415. doi: 10.3390/mi12040415 (PMC8069467; doi:10.3390/mi12040415)
Supplement: Supplementary file 1 [file micromachines-12-00415-s001.pdf]

micromachines

Supplementary Material

1. Fabrication process of DDM.

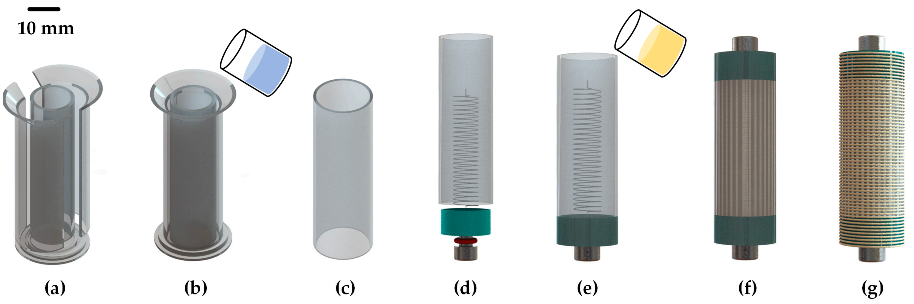

Figure S1. Fabrication process of DDM. (**a**) Printing using a 3D printer and assembly of the mold of silicone rubber shell. (**b**) Mixing, pouring, and curing after vacuum of silica gel components A and B. (**c**) Demolding of silicone rubber shell. (**d**) Placement of heating wire and other components. (**e**) Pouring and curing of PCMs under normal temperature. (**f**) Packaging of the upper end cover. (**g**) Formation of the restricted layer through the close winding of aramid fiber.

2. Response time of DDM in the air.

Table S1. Response time of DDM in the air.

Test environment	Power (W)	Electrification time (s)	Average retraction time (s)
Air	30	211	3000
	40	163	
	50	128	
	60	109	
	70	91	
	80	82	
	90	72	

3. Response time of DDM underwater.

Table S2. Response time of DDM underwater.

Test environment	Power (W)	Electrification time (s)	Average retraction time (s)
Underwater	50	256	190
	60	181	
	70	149	
	80	118	
	90	100	

4. In the pressure chamber, a set of preparatory experiments at 17.8 °C and 10 MPa hydrostatic pressure.

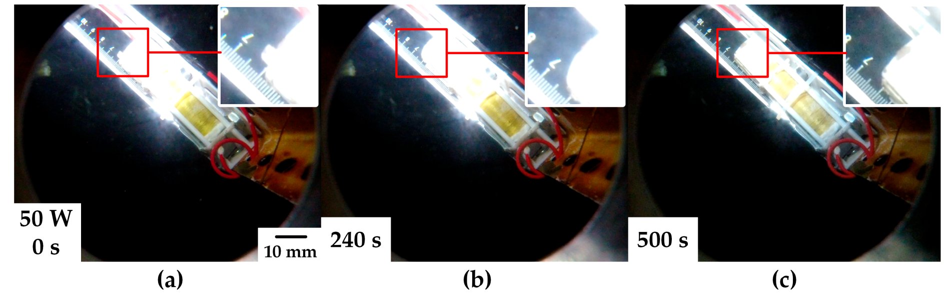

Figure S2. DDM test at 10 MPa hydrostatic pressure: (**a**) original state, (**b**) operating state at 50 W power, and (**c**) end state.
